# The impact of water safety skills on primary school students high-risk swimming behaviors: a moderated mediation model

**DOI:** 10.3389/fpubh.2025.1639456

**Published:** 2025-09-17

**Authors:** Zhen Tan, Jiaxin Shi, Chao Xie, Hui Zhang

**Affiliations:** School of Physical Education, Hubei Minzu University, Enshi, China

**Keywords:** high-risk swimming behavior, water safety skills, self-efficacy, water safety knowledge, primary school student

## Abstract

**Introduction:**

To investigate the relationship between water safety skills and high-risk swimming behaviors and the underlying mechanisms of self-efficacy and water safety knowledge.

**Methods:**

Water safety skills, self-efficacy, water safety knowledge, and high-risk swimming behaviors were investigated in 1573 primary school student using a questionnaire.

**Results:**

(1) After controlling for gender, age, and grade, there was a significant positive effect of water safety skills on high-risk swimming behaviors [*β* = 0.062, 95%CI (0.037, 0.088), *p* < 0.01]; (2) self-efficacy partially mediated the relationship between water safety skills and high-risk swimming behavior [*β* = 0.050, 95%CI (0.039, 0.060), *p* < 0.01]; and (3) the mediating role of self-efficacy in the relationship between water safety skills to high-risk swimming behaviors was negatively moderated by knowledge of water safety [*β* = −0.026, 95%CI (−0.035, −0.016), *p* < 0.01].

**Conclusion:**

Self-efficacy mediates between water safety skills and high-risk swimming behavior; water safety knowledge moderates between water safety skills and high-risk swimming behavior.

## 1 Introduction

Drowning, as a major global public health problem, continues to receive international attention. According to the World Health Organization Global Drowning Report 2021, drowning causes about 300,000 deaths per year, which is equivalent to more than 34 deaths per hour, and primary school student with high-risk behavioral characteristics of swimming are particularly at high risk (1). The Ministry of Education’s ‘2022 China Youth Drowning Prevention Data Report’ points out that about 59,000 people die from drowning every year, of which 56.7% are aged 0–17, and the drowning rate of primary school student aged 1–14 exceeds 40% (2), indicating that drowning has become the ‘number one killer’ of primary school student accidental deaths. The data show that drowning has become the ‘number one killer’ of unintentional deaths among primary school student. Although water safety skills are generally regarded as a key intervention to prevent drowning, the potential ‘double-edged sword effect’ gradually emerges among primary school student with uneven skill mastery, which may induce overconfidence and increase the incidence of risky behaviors while improving skill levels (3). Therefore, the psychological impact mechanism of water safety skills on primary school students has gradually become the focus of researchers’ attention. Existing studies have demonstrated that water safety skills are significantly associated with self-protection (4), emotion regulation (5), and risk assessment accuracy (6), especially in predicting self-efficacy (7) and the tendency to engage in high-risk swimming behaviors (8), which has become an important issue in public health research.

High-risk swimming behavior refers to the risky behaviors of individuals in open or non-open water environments that are likely to cause harm to themselves, which may hurt the physical and mental health of individuals and may even lead to irreversible damage. Therefore, academics have explored the mechanisms of the emergence and development of high-risk behavior in swimming from multiple dimensions. Studies have found that high-risk swimming behaviors are driven by subjective factors such as endogenous traits (8), cognitive biases (9), and knowledge deficits (10), as well as exogenous environmental factors (11). Studies have also shown that high skills are important triggers for high-risk behaviors (12), but the relationship between water safety skills and high-risk swimming behaviors, as well as how and when water safety skills influence high-risk swimming behaviors of primary school students, still needs to be further explored. In recent years, the academic community has held that the emergence of high-risk swimming behaviors among primary school students may be the result of the combined effect of multiple factors, and the relationship between water safety skills and high-risk swimming behaviors may be influenced by various factors (13, 14). Therefore, to further clarify the formation and development mechanism of high-risk swimming behaviors among primary school students, it is necessary to examine the mediating and moderating effects of ability factors (water safety skills), psychological factors (self-efficacy), and cognitive factors (water safety knowledge) on high-risk swimming behaviors among primary school students from the perspective of multi-factor integration. According to the self-efficacy theory, an individual’s ability beliefs have a significant impact on their risk perception and corresponding action strategies. Therefore, an individual’s cognitive bias toward their own abilities may become a key psychological factor that triggers high-risk behaviors (15). Some research suggests that self-efficacy might be a mediating variable in explaining how other factors ‘influence’ individual behavior (16). Therefore, studying the mediating role of self-efficacy is helpful to reveal the cognitive mechanism by which water safety skills influence high-risk swimming behaviors among primary school students. In addition, water safety knowledge acts as a moderating variable to buffer the strength of the influence of other risk factors during risky behavior. The protection motivation theory also points out that the psychological activities and behavioral regulation of individuals when assessing and responding to risks are jointly influenced by several psychological factors, among which self-efficacy is particularly crucial. Adequate safety knowledge reserves are the key factors in suppressing the occurrence of high self-efficacy and high-risk behaviors of individuals (17). Therefore, the introduction of water safety knowledge as a moderating variable can help to reveal individual differences in self-efficacy leading to high-risk behaviors in swimming.

Based on this, the study integrates self-efficacy and protective motivation theories to explore the relationship between water safety skills and high-risk swimming behavior and its underlying mechanisms, focusing on the mediating role of self-efficacy and the moderating effect of water safety knowledge. In this way, the study will answer the question of ‘how’ water safety skills affect high-risk swimming behavior and ‘when the effect decreases’.

Water safety skills refer to an individual’s ability to prevent accidents, respond to emergencies, and ensure the safety of themselves and others in a water environment. A follow-up study by Xia Wen based on the KSAP (Knowledge, waters, attitude, performance) model found that improved water safety skills did not produce risk prevention and control as expected, but instead may enhance primary school student risk perception bias, which in turn leads to an increased probability of risk-taking behaviors (18). The study concluded that water safety skills have a significant impact on an individual’s psychological and behavioral competence and induce high-risk behaviors in swimming. At the individual level, although individuals with higher water safety skills theoretically have a lower probability of engaging in high-risk swimming behaviors, empirical data verified that high-skilled individuals are more inclined to actively expose themselves to hazardous waters or attempt high-risk maneuvers, which can lead to drowning incidents (19). This phenomenon is in line with the ‘ability-risk paradox’, i.e., the ancient Chinese saying: ‘Those who are good at riding fall off their horses and those who are good at water drown in water’, which reveals the potential correlation between skill proficiency and weakened risk perception. At the environmental level, lax parental supervision due to higher skills possessed by their primary school student, coupled with the risk-normalizing tendency of undesirable peers, can weaken individual risk judgment and increase the prevalence of high-risk behaviors in swimming. The model of factors influencing high-risk behavior in swimming also points to skill level and environmental conditions as key variables contributing to high-risk behavior in swimming (8). Empirical studies have further confirmed that water safety skills have a significant positive effect on high-risk behavior in swimming (20). In summary, Hypothesis H1: Water safety skills positively influence high-risk behavior in swimming is proposed.

Self-efficacy refers to an individual’s subjective beliefs about accomplishing a specific goal or task, and the stronger the beliefs the stronger the individual’s motivation to behave in complex environments (21). Self-efficacy can be affected by a variety of factors such as individual risk perception, behavioral motivation, emotion regulation, overconfidence, and so on. Among them, the level of self-confidence and water safety skills are also important factors affecting self-efficacy. Research based on the level of self-confidence suggests that overconfidence may lead to an irrational expansion of individual self-efficacy, which is manifested by overestimation of one’s ability and underestimation of the potential risk in the face of risky behaviors (22). Research has also concluded that developing water safety skills increases an individual’s self-confidence, which significantly increases self-efficacy (7). Therefore, water safety skills may positively influence individual self-efficacy. It has been established that self-efficacy has a significant positive effect on high-risk swimming behavior (23). Moreover, self-efficacy theory also points out that high self-efficacy triggered by psychological and behavioral factors is an important cause of blunted risk perception and high-risk behaviors in individuals (15). Therefore, Hypothesis H2: Water safety skills have an indirect effect on high-risk behavior in swimming through the mediating role of self-efficacy is proposed.

Water safety knowledge, which refers to procedural knowledge about swimming, self-rescue, and drowning rescue, is a key moderating variable for individuals to inhibit overconfidence and reduce the incidence of risky behaviors. Research has shown that water safety knowledge achieves its effects mainly by modulating individuals’ cognitive mechanisms and risk judgments. From the perspective of cognitive mechanisms, when faced with environmental stimuli or interference from psychological factors, individuals with adequate water safety knowledge can implement defensive behavioral strategies more effectively and mitigate the negative influence of psychological factors (e.g., self-efficacy) on high-risk swimming behavior, whereas individuals with inadequate water safety knowledge are more susceptible to interference or temptation from environmental factors (19), which exacerbates the influence of psychological factors, which to some extent exacerbates the negative influence of psychological factors (e.g., self-efficacy) on their high-risk behavior in swimming. From the perspective of risk judgment, individuals with adequate knowledge of water safety tend to have higher risk judgment ability, which is conducive to individuals inhibiting the interference of environmental factors and reducing the occurrence of high-risk swimming behaviors. Individuals with poor water safety knowledge, on the other hand, have weaker risk judgment ability, are more likely to unconsciously engage in risky behaviors, and to a certain extent increase the likelihood of self-efficacy-induced high-risk swimming behaviors. In addition, the theory of protective motivation also points out that when influenced by psychological factors (e.g., self-efficacy), the failure of relevant knowledge to self-regulate the individual’s psychological and behavioral activities is a key factor leading to the development of high-risk behaviors (17). Accordingly, Hypothesis H3 has proposed: that knowledge of water safety plays a negative moderating role in the relationship between self-efficacy and high-risk behavior in swimming.

**Figure 1:**
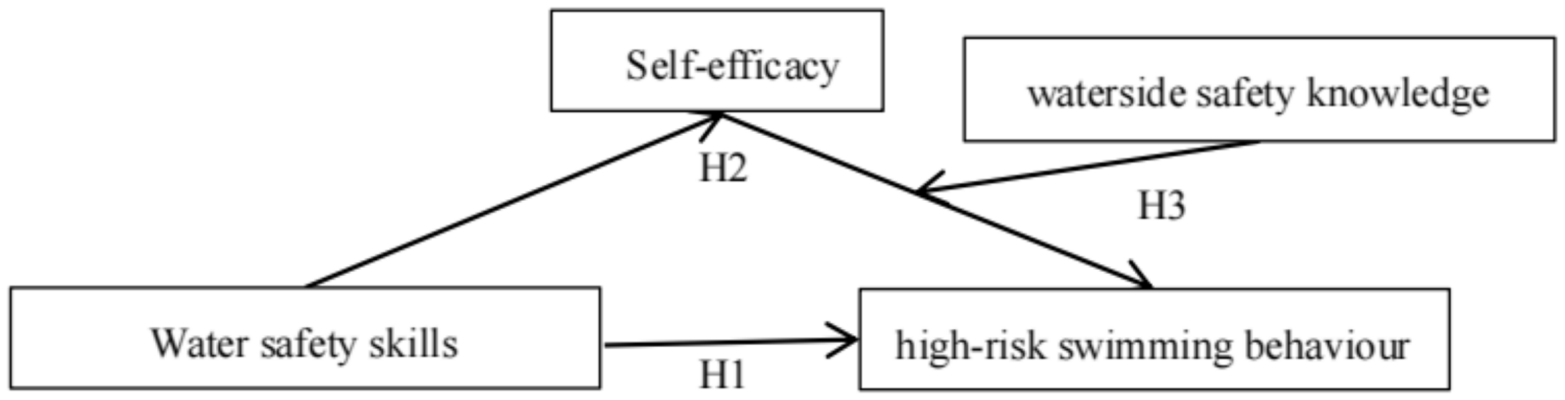
Diagram of the hypothetical model.

Given this, based on the integration of self-efficacy theory and protection motivation theory, a hypothetical model of the influence of water safety skills on high-risk swimming behaviors was constructed (Figure 1) and simultaneously examines the relationships between water safety skills, self-efficacy, and knowledge of water safety and primary school student high-risk swimming behaviors. Specifically, the study examined the mediating (self-efficacy) and moderating (water safety knowledge) mechanisms through which water safety skills affect primary school student high-risk swimming behaviors, to identify the psychological factors and individual differences in water safety skills that lead to high-risk swimming behaviors in primary school student, and providing theoretical guidance and practical support for more targeted guidance to primary school student in addressing their water safety skills, and in avoiding or reducing high-risk swimming behaviors.

## 2 Materials and methods

### 2.1 Participants and procedure

The cluster sampling method was employed. Two urban and two rural primary schools in Hubei Province were selected as survey sites, and students from grades three to six were included in the sample, to ensure sample diversity and data validity. A total of 1,698 questionnaires were distributed, and 1,573 valid questionnaires (92.64%) were obtained after recovery and collation. The average age of the subjects was 11.32 ± 0.49 years old, of which 850 (54%) were boys and 723 (46%) were girls, 999 (63.5%) were in Grade 5, and 574 (36.5%) were in Grade 6. The study was approved by the Ethics Committee of Hubei Minzu University (ethical approval number: 101).

### 2.2 Measures

#### 2.2.1 Water Safety Skills and Knowledge Scale

The Student Water Safety Knowledge, Belief and Behavior Scale developed by Xia Wen was used to assess the level of water safety skills of students (24). The study selected the water safety skills and knowledge sub-questionnaire as the measurement tool, which included a total of 19 items and was scored on a scale of 1 to 5 (1 = very unfamiliar, 5 = very familiar). The total score of water safety skills and knowledge of the subject is the sum of the scores of all items. The higher the score, the higher the level of skills and knowledge. The Cronbach’s *α* coefficients of the Water Safety Skills and Knowledge Scale were 0.964 and 0.963 respectively, indicating that the scale has good internal consistency. In addition, this scale is closely integrated with China’s water safety culture and the actual situation of primary school student, enhancing the applicability and understandability of the questionnaire. However, compared with international tools, it may have limitations in cross-cultural applications.

#### 2.2.2 Self-Efficacy Scale

The Swimming Self-Efficacy Scale developed by Theodorakis was used to measure the strength of subjects’ perceptions of their level of swimming skills (25). The scale consists of 6 entries and is rated on a scale of 1–10 (1 = not at all, 10 = very sure). The scores of all the entries were summed up to be the total self-efficacy score of the subjects, with higher scores indicating stronger self-efficacy of the individual. The Cronbach’s alpha coefficient for the Self-Efficacy Scale in this measurement was 0.850, indicating that the scale has good internal consistency.

#### 2.2.3 Swimming Risk Behavior Scale

The self-administered Swimming Risk Behavior Scale was used to assess adolescents’ swimming risk behavior. The scale consists of 20 entries and is rated on a scale of 1–5 (1 = strongly agree, 5 = strongly disagree). To prevent primary school student from answering consistent questions, all items are encoded in reverse. The higher the score, the lower the frequency of an individual’s participation in high-risk swimming behaviors. The scale had a Cronbach’s *α* coefficient of 0.859, a fit index of χ^2^/df = 3.672, RM*R* = 0.048, TLI = 0.957, CFI = 0.938, and RMSEA = 0.045, indicating good reliability and validity.

### 2.3 Measurement procedure

Informed consent was obtained from the Education Bureau of Enshi Prefecture, Hubei Province, school management, classroom teachers, parents, and the students themselves before the tests were officially administered. The administration was organized on a class-by-class basis to ensure efficiency and orderliness. All questionnaires were completed anonymously to collect demographic information including gender, age, grade level, and four scales with special attention to the reverse scoring design for some items. Once the collection of questionnaires was completed, invalid questionnaires were excluded, the data were analyzed.

### 2.4 Data processing

The study used AMOS 28.0 software for common method bias test, and validation factor analysis; then SPSS 28.0 software for descriptive statistics and correlation analysis; and finally PROCESS 4.1 developed by Hayes for mediation and moderated effects analysis (26).

## 3 Results

### 3.1 Control and testing of common method deviations

To address the potential common method bias, the study introduced the ‘control of untested single-method latent factor method’ to test the common method bias based on the implementation of procedural controls such as anonymized responses and reverse coding of some entries. First, a validated factor analysis model M1 was constructed; second, a model with method factors was constructed M2, and the main fit indices of the two models were compared, which were: ΔCFI = 0.004, ΔTLI = 0.014, ΔNFI = 0.018, and ΔRMSEA = 0.004. The changes in each fitting index were all less than 0.02, indicating that the model did not improve significantly after adding the common method factor. Therefore, it is considered that there is no obvious common method bias in the measurement (27).

### 3.2 Mean, standard deviation and correlation matrix for each variable

The results of the descriptive and correlational analyses showed (Table 1) that water safety skills (*r* = 0.13, *p* < 0.01) and self-efficacy (*r* = 0.23, *p* < 0.01) were significantly and positively correlated with high-risk swimming behavior, suggesting that all are risk factors for primary school student high-risk swimming behavior. Water safety knowledge (*r* = −0.07, *p* < 0.01) was significantly negatively correlated with high-risk swimming behavior, indicating that water safety knowledge may be a protective factor for students’ high-risk swimming behavior.

**Table 1:** Results of descriptive statistics, correlation analysis.

Variable	M	SD	Water safety skills	Self-efficacy	Water safety knowledge	High-risk swimming behavior
Water safety skills	2.43	1.19	1			
Self-efficacy	2.76	2.30	0.32**	1		
Water safety knowledge	3.46	1.09	0.44**	0.12**	1	
High-risk swimming behavior	1.53	0.54	0.13**	0.23**	−0.07**	1

**P < 0.01.

### 3.3 Moderated mediation model test

Firstly, the mediating effect of self-efficacy between water safety skills and high-risk behavior in swimming was examined using Model 4 (Simple Mediation Model) (26), an SPSS macro program developed by Hayes, controlling for the variables of gender, age and grade. As shown in Tables 2, 3, the effect of water safety skills on high-risk swimming behavior was significant (*β* = 0.060, *t* = 5.237, *p* < 0.01), and the direct effect of water safety skills on high-risk swimming behavior remained significant after putting in the mediating variables (*β* = 0.030, *t* = 2.546, *p* < 0.01). The positive effect of water safety skills on self-efficacy was significant (*β* = 0.615, *t* = 13.251, *p* < 0.01), as was the positive effect of self-efficacy on high-risk swimming behavior (*β* = 0.049, *t* = 7.933, *p* < 0.01). In addition, the direct effect of water safety skills on high-risk swimming behavior and the mediating effect of self-efficacy did not include 0 in the upper and lower bounds of Bootstrap 95% confidence intervals, and the direct effect (0.030) and the mediating effect (0.030) accounted for 50% of the total effect (0.060), which indicated that water safety skills not only directly affected high-risk swimming behavior, but also influenced high-risk swimming behavior through the mediating effect of self-efficacy. Hypotheses H1 and H2 can be verified.

**Table 2:** Mediation model test for self-efficacy.

Regression equation (*N* = 1,573)		Fitness index			Significance of coefficients	
Outcome variable	Influencing variable	R	R^2^	F(df)	*β*	t
High-risk swimming behavior		0.147	0.022	8.673**		
	Genders				−0.040	−1.458
	Age				0.069	1.901
	Grade				−0.025	−0.671
	Water safety skills				0.060	5.237**
Self-efficacy		0.322	0.104	45.350**		
	Genders				−0.161	−1.455
	Age				0.075	0.512
	Grade				−0.046	−0.309
	Water safety skills				0.615	13.251**
High-risk swimming behavior		0.244	0.059	19.798**		
	Genders				−0.032	−1.195
	Age				0.065	1.835
	Grade				−0.023	−0.622
	Self-efficacy				0.049	7.933**
	Water safety skills				0.030	2.546*

**Table 3:** Analysis of total effects, direct effects and mediating benefits.

Variable relationship	Efficiency value	Boot standard error	BootCI lower limit	BootCI upper limit	Relative effect value
Aggregate effect	0.060	0.012	0.037	0.082	
Direct effect	0.030	0.012	0.007	0.053	50%
Mediating effects of self-efficacy	0.030	0.006	0.018	0.043	50%

**Figure 2:**
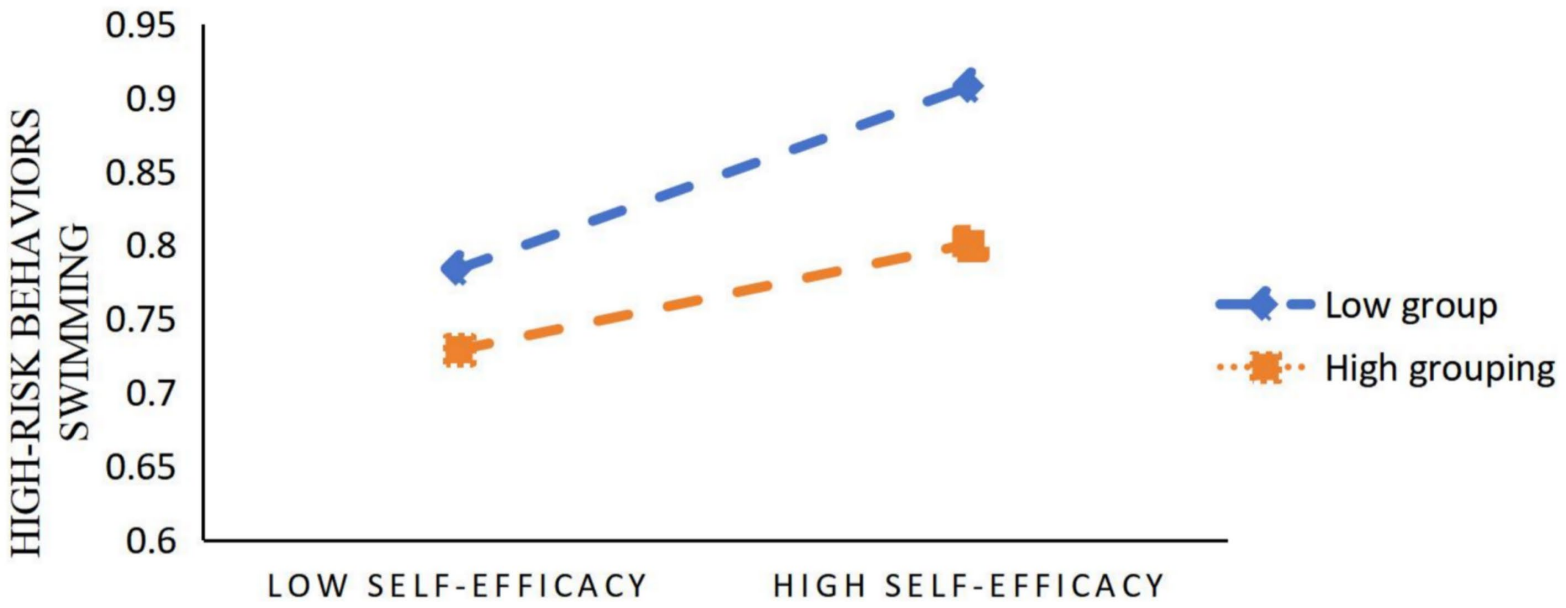
Negative moderating effect of water safety knowledge on self-efficacy on high-risk swimming behaviors.

Next, Model 14 (Moderated Mediation Model) in the SPSS macro program prepared by Hayes was used to test the moderating effect of water safety knowledge on the mediating pathway of self-efficacy, controlling for gender, age, and grade variables. As shown in Tables 4, 5, the product term of self-efficacy and water safety knowledge had a significant effect on high-risk swimming behaviors after placing water safety knowledge into the model (*β* = −0.026, *t* = −5.439, *p* < 0.01). The results of the negative regulation of self-efficacy by water safety knowledge on high-risk swimming behaviors show (Figure 2) that for primary school students (M-SD) with a lower level of water safety knowledge, the positive effect of self-efficacy on high-risk swimming behaviors is significant (*β* = 0.079, *t* = 9.53, *p* < 0.01); For primary school students (M + SD) with a relatively high level of water safety knowledge, although self-efficacy also has a positive impact on high-risk swimming behaviors, its positive impact is weakened (*β* = 0.023, *t* = 2.97, *p* < 0.01), indicating that with the improvement of primary school students’ water safety knowledge level, The influence of self-efficacy on high-risk swimming behaviors shows a gradually decreasing trend. In addition, the mediating effect of self-efficacy in the relationship between water safety skills and high-risk swimming behaviors also tended to decrease at the three levels of water safety knowledge, which means that water safety skills are less likely to induce high-risk swimming behaviors by increasing the self-efficacy of the elementary school students through increasing the self-efficacy of the subjects as their knowledge of water safety becomes gradually more abundant, and Hypothesis H3 is thus verified.

**Table 4:** Mediation model test with moderation.

Regression equation (*N* = 1,573)		Fitness index			Significance of coefficients	
Outcome variable	Influencing variable	R	R^2^	F(df)	*β*	t
High-risk swimming behavior		0.307	0.095	23.325**		
	Genders				−0.019	−0.731
	Age				0.062	1.778
	Grade				−0.024	−0.673
	Self-efficacy				0.505	8.391**
	Water safety knowledge				−0.081	−0.599**
	Self-efficacy × Water safety knowledge				−0.026	−5.439**

**Table 5:** Mediating effects at different levels of water safety knowledge.

Mediating effect	Water safety knowledge	Efficiency value	Boot standard error	BootCI lower limit	BootCI upper limit
The mediating role of self-efficacy	M-SD	0.048	0.012	0.026	0.073
	M	0.031	0.007	0.019	0.044
	M + SD	0.014	0.007	0.001	0.029

## 4 Discussion

Based on the self-efficacy theory and the protection motivation theory, a moderated mediation model was constructed with self-efficacy as the mediating variable and water safety knowledge as the moderating variable. The research results show that there is a significant direct effect between water safety skills and high-risk swimming behaviors. Self-efficacy plays a partial mediating role between water safety skills and high-risk swimming behaviors. Water safety knowledge, as an important protective factor for primary school students, exerts a negative moderating effect. It is worth noting that water safety skills have a paradoxical impact on primary school students: on the one hand, they significantly enhance an individual’s ability to deal with water risks by providing swimming, self-rescue and drowning rescue techniques, and play an important protective role; On the other hand, if one only focuses on swimming techniques while neglecting self-rescue and drowning rescue skills, it may induce overconfidence and increase high-risk behaviors [29]. The study starts with high-risk swimming behaviors and discusses the impact of skill levels, self-efficacy and water safety knowledge on high-risk behaviors. Therefore, the focus of the study is on the latter. So, how does a higher skill level lead to an increase in high-risk behaviors? What are the specific impacts on schools, parents and water safety training as the higher the swimming skills of primary school students, the more high-risk swimming behaviors they exhibit?

The reason for the previous question is that individuals with higher water safety skills participate in water activities more frequently and experience more diverse water environment stimuli, which may stimulate their desire for more difficult challenges and increase risks. The positive reinforcing effect of water safety skills on self-efficacy further confirms the ‘ability-risk paradox’, that is, high skill levels may, by influencing an individual’s risk perception bias (such as underestimating risks), instead lead to the occurrence of high-risk swimming behaviors. Empirical cases strongly support this paradox: for instance, a child with swimming skills in Guangzhou, China, accidentally drowned while competing with his peers in underwater swimming, highlighting that even those with proficient skills may encounter misfortune due to insufficient risk awareness or misjudgment of the situation. In addition, in the high-incidence waters of wild swimming in Changsha City, China, there are often cases where people and children ignore warning signs and enter the water. Their common mentality of ‘I have been swimming for many years without any problem, and the risk has nothing to do with me’ is a typical manifestation of overconfidence and has led to many drowning tragedies. In the face of the second issue, the phenomenon that the improvement of primary school students’ water safety skills is accompanied by an increase in high-risk swimming behaviors poses new challenges and requirements for parents, schools and water safety training. For parents, this phenomenon provides an important warning that the improvement of water safety skills among primary school students does not necessarily mean they are safer; instead, it may increase high-risk swimming behaviors due to self-efficacy (such as overconfidence). Therefore, parents need to actively participate in the water safety education process for primary school students. They should not only pay attention to the progress of skills training but also attach importance to the imparting and internalization of safety knowledge. They should adjust their expectations and evaluation standards for primary school students’ swimming abilities from ‘how far they can swim’ to ‘how to swim safely’, and enhance their own water safety knowledge level to more effectively guide and supervise their children’s water activities. For schools, this phenomenon challenges the traditional concept of “skills first” in water safety education, and it is necessary to re-examine the curriculum design to achieve a comprehensive education model that emphasizes both skills and knowledge. Water safety knowledge education should be elevated to the same level of importance as water safety skills. Risk awareness education, self-efficacy management, and methods and tools to assist in skill learning should be introduced into the curriculum to help primary school students establish accurate ability assessment and risk judgment mechanisms and reduce high-risk behaviors among them (28). At the same time, the teacher training system also needs to be updated accordingly. For instance, the latest information can be adopted to enable educators to understand the complex interaction among skills, knowledge and self-efficacy, thereby effectively guiding students in teaching to avoid overconfidence caused by the improvement of water safety skills. For water safety training, this phenomenon requires a rethinking of training concepts and methods, shifting from the traditional skills-centered model to a comprehensive training model of ‘skills+ knowledge+ risk awareness’. The training course structure needs to be redesigned to ensure the organic integration of water safety knowledge and skills training. During the training process, the self-efficacy of primary school students should be consciously managed to prevent overconfidence caused by skill improvement. In addition, the professional development of coaches also needs to be adjusted to enable them to master how to balance the improvement of skills and the cultivation of risk awareness in the teaching process, and be able to identify and correct the possible overconfidence tendencies of primary school students.

In view of this, families, schools and training institutions need to form an educational synergy to jointly pay attention to the all-round development of primary school students’ water safety, and avoid the cancellation of safety education effects caused by differences in educational concepts and methods among all parties. Only in this way can the high-risk swimming behaviors of primary school students be truly and effectively reduced, and their water activity safety be guaranteed.

## 5 Conclusion

(1) Water safety skills positively affect high-risk swimming behaviors of primary school students, that is, the irrational use of water safety skills leads to a higher likelihood of high-risk swimming behaviors of primary school students; (2) Self-efficacy plays a partial mediating role in the influence path of water safety skills on high-risk swimming behaviors of primary school students, that is, water safety skills can not only directly and positively affect high-risk swimming behaviors of primary school students, but also indirectly affect high-risk swimming behaviors of primary school students through self-efficacy, which is specifically manifested in the positive impact of water safety skills on self-efficacy, self-efficacy positively affects the high-risk swimming behavior of primary school students; (3) Water safety knowledge negatively regulates self-efficacy in the latter half of the mediating process between water safety skills and high-risk swimming behaviors of primary school students, that is, the influence of self-efficacy on high-risk swimming behaviors of primary school students is negatively regulated by water safety knowledge, which is specifically manifested as: The high risk swimming behavior of primary school students with abundant water safety knowledge is significantly lower than that of primary school students with poor water safety knowledge.

## 6 Advantages and limitations

Advantages: (1) By comprehensively applying the Water Safety Skills and Knowledge Scale, the Self-Efficacy Scale, and the Swimming High-Risk Behavior Scale, the water safety skills, self-efficacy, water safety knowledge, and swimming high-risk behaviors of primary school students were comprehensively evaluated, providing a solid foundation for in-depth analysis of the relationships among various variables; (2) The research adopted advanced statistical methods, such as confirmatory factor analysis, mediating effect and moderating effect analysis, strictly controlling potential interfering factors such as common method bias, ensuring the accuracy and validity of the research results. (3) The research has revealed the complex relationship between water safety skills and high-risk swimming behaviors, especially the mediating role of self-efficacy, providing a new perspective for understanding the psychological mechanism of high-risk swimming behaviors among primary school students.

Limitations: (1) There are limitations in the geographical distribution of the research samples. Future research should expand the sample range to include children of different age groups and from different regional backgrounds to enhance the universality of the research conclusions. (2) Current research focuses on high-risk swimming behaviors of primary school students at the individual level. Future research can be expanded to the group or community level to explore the interaction of different levels of factors (such as peer influence and community norms) on high-risk behaviors. (3) The research mainly examined the moderating role of water safety knowledge. Future studies can delve into more complex moderating or mediating mechanisms between water safety skills and high-risk swimming behaviors in children from other dimensions, such as risk perception, self-efficacy, and bad peers.

## Data Availability

The original contributions presented in the study are included in the article/Supplementary material. Further inquiries can be directed to the corresponding author.
